# Evolution and Genetic Architecture of Chromatin Accessibility and Function in Yeast

**DOI:** 10.1371/journal.pgen.1004427

**Published:** 2014-07-03

**Authors:** Caitlin F. Connelly, Jon Wakefield, Joshua M. Akey

**Affiliations:** 1 Department of Genome Sciences, University of Washington, Seattle, Washington, United States of America; 2 Department of Statistics, University of Washington, Seattle, Washington, United States of America; University of Michigan, United States of America

## Abstract

Chromatin accessibility is an important functional genomics phenotype that influences transcription factor binding and gene expression. Genome-scale technologies allow chromatin accessibility to be mapped with high-resolution, facilitating detailed analyses into the genetic architecture and evolution of chromatin structure within and between species. We performed Formaldehyde-Assisted Isolation of Regulatory Elements sequencing (FAIRE-Seq) to map chromatin accessibility in two parental haploid yeast species, *Saccharomyces cerevisiae* and *Saccharomyces paradoxus* and their diploid hybrid. We show that although broad-scale characteristics of the chromatin landscape are well conserved between these species, accessibility is significantly different for 947 regions upstream of genes that are enriched for GO terms such as intracellular transport and protein localization exhibit. We also develop new statistical methods to investigate the genetic architecture of variation in chromatin accessibility between species, and find that *cis* effects are more common and of greater magnitude than *trans* effects. Interestingly, we find that *cis* and *trans* effects at individual genes are often negatively correlated, suggesting widespread compensatory evolution to stabilize levels of chromatin accessibility. Finally, we demonstrate that the relationship between chromatin accessibility and gene expression levels is complex, and a significant proportion of differences in chromatin accessibility might be functionally benign.

## Author Summary

Inside the nucleus of a cell, DNA is associated with proteins to form a complex three-dimensional structure referred to as chromatin. The structure of chromatin influences how accessible specific DNA sequences are to transcription factors, and therefore chromatin accessibility is an important determinant of gene expression. To better understand how patterns of chromatin accessibility change over time, we quantitatively measured levels of chromatin accessibility in two yeast species and their diploid hybrid. We show that significant differences in chromatin accessibility exist between these two species and occur upstream of genes that are enriched for specific biological functions. We also develop new statistical methods to understand the genetics of variation in chromatin accessibility. Finally, we show that the relationship between chromatin accessibility and gene expression is complex, and many of the observed differences in chromatin accessibility between these two species may not influence gene expression levels. Thus, our work highlights the need to develop additional experimental and statistical methods to distinguish between functionally significant and benign changes in chromatin accessibility.

## Introduction

Changes in gene regulation have long been hypothesized to be an important mechanism of evolutionary diversification [1]–[3] and to contribute to phenotypic variation [4]–[7]. An increasing catalog of adaptive regulatory changes has been identified, such as lactase persistence [8], [9] and the effect of the Duffy blood group chemokine receptor on malaria resistance in humans [10], [11] and beak morphology in Darwin's finches [12]. Furthermore, it has also been suggested that a substantial fraction of SNPs associated with human diseases through genome-wide association studies may act through regulatory changes with genes [13], [14].

On a genome-wide scale, molecular studies have uncovered pervasive transcriptional variation within and between species [15]–[20]. A substantial amount of gene expression variation is heritable, and thousands of regulatory QTL have been mapped in numerous organisms [17], [21]–[24]. In general, regulatory variation can act in *cis* or *trans*. *Cis*-acting regulatory QTL influence transcript levels in an allele-specific manner, typically from variation located within or near the gene being studied. In contrast, *trans*-acting regulatory QTL does not result in allelic differences in expression and arises from variation that is usually located at a position distinct from the gene being studied [7]. Although both *cis* and *trans* regulatory variation make important contributions to heritable variation of transcript abundance, *cis*-acting variants are thought to be more numerous, have larger effect sizes, and accumulate at a faster rate between species [21], [25].

Despite the progress in mapping *cis* and *trans*-acting regulatory QTL, the mechanisms they act through are less well understood. Chromatin structure is a fundamentally important determinant of gene regulation, and changes in the position and number of nucleosomes can affect transcript abundance [26]–[29]. New technologies have enabled genome-wide maps of chromatin architecture to be constructed across different cell types [30], [31] individuals [32]–[34] and species [20], [35]. Although these studies have revealed extensive variation in chromatin structure, many outstanding issues remain, including how much of variation in chromatin accessibility is heritable, the relative contributions of *cis* and *trans*-acting regulatory variation to differences in chromatin architecture [32], and how often variation in chromatin structure results in gene expression variation [22], [36].

To address these issues, we describe a genome-wide analysis of chromatin accessibility between two closely related *Saccharomyces sensu stricto* yeast species, *Saccharomyces cerevisiae* and *Saccharomyces paradoxus*, and their hybrid. *S. cerevisiae* is the yeast model species and has been extensively studied. *S. paradoxus* is the most closely related species to *S. cerevisiae*, with an estimated divergence time of 5 million years ago [37]. Chromatin structure in *S. cerevisiae* has been studied previously [38], [39] and across a single genome, open chromatin regions are weakly associated with increased expression [39]. In addition, nucleosome locations have been compared across multiple yeast species, including *S. cerevisiae* and *S. paradoxus*, and *cis* changes, such as anti-nucleosomal sequences and binding sites for general regulatory factors, were found to contribute to differences in nucleosome location [20]. Within species, the genetic architecture of chromatin accessibility has been studied using QTL mapping [34]; however, this has not been addressed between species.

We assessed chromatin accessibility using FAIRE-Seq and found considerable divergence in chromatin structure between *S. cerevisiae* and *S. paradoxus*. Moreover, we developed a novel statistical approach to identify *cis* and *trans*-acting effects on chromatin accessibility in hybrids and found *cis* effects on chromatin structure are more common than *trans* effects, are of greater magnitude, and that the direction of *cis* and *trans* effects are often in opposite directions suggesting compensatory evolution. Finally, we show that the relationship between chromatin structure and transcript levels in *S. cerevisiae* and *S. paradoxus* is complex, and a significant proportion of differences in chromatin accessibility might be functionally benign.

## Results

### Differences in chromatin accessibility within and between species

We first assessed differences in chromatin structure between haploid strains of *S. cerevisiae* and *S. paradoxus*. We generated FAIRE-Seq (Formaldehyde-Assisted Isolation of Regulatory Elements) data [40] for two biological replicates for two strains of *S. cerevisiae* (DBVPG1373, a wine strain, and UWOPS05_217_3, a wild isolate) and one strain of the sister species *S. paradoxus*, CBS432 (see Methods). FAIRE isolates DNA that is not bound to proteins, resulting in increased signal in regions with increased chromatin accessibility. We sequenced FAIRE DNA samples to approximately 10× coverage using short read sequencing (see Methods). As expected, sequencing reads were enriched in intergenic regions (mean of 2.4× enrichment compared to coding regions).

We first asked which specific areas of the genome have undergone changes in chromatin accessibility between species. We focused on the nucleosome-free region (NFR) found upstream of the transcription start site of many yeast genes because this region is known to harbor important regulatory information; this was also where the dominant FAIRE signal was found in our data [41], (see Figure S1). We computationally identified the nucleosome-free region from the FAIRE data (see Methods) by identifying the peak in FAIRE signal found upstream of each gene and extended the region in either direction until a background level of signal was observed. We then merged NFR calls across the two species (see Methods). We also carried out extensive filtering to eliminate peaks whose differences might be caused by duplications between species or mapping issues (see Methods). In total, we identified 3,498 NFRs that passed our filtering and had an average size of 253 bp.

We first compared one strain of *S. paradoxus*, CBS432, and one strain of *S. cerevisiae*, UWOPS05_217_3. Overall, the locations of NFRs called were well-conserved across species, and on average the location of 42% of NFRs overlapped between the two species. As a complementary analysis, we compared levels of chromatin accessibility in the set of all 3,498 NFRs, and found them to be strongly correlated (R^2^ = 0.68 between species, *p*<2.2×10^−16^) suggesting that broad-scale patterns of accessibility are conserved over time.

**Figure 1:**
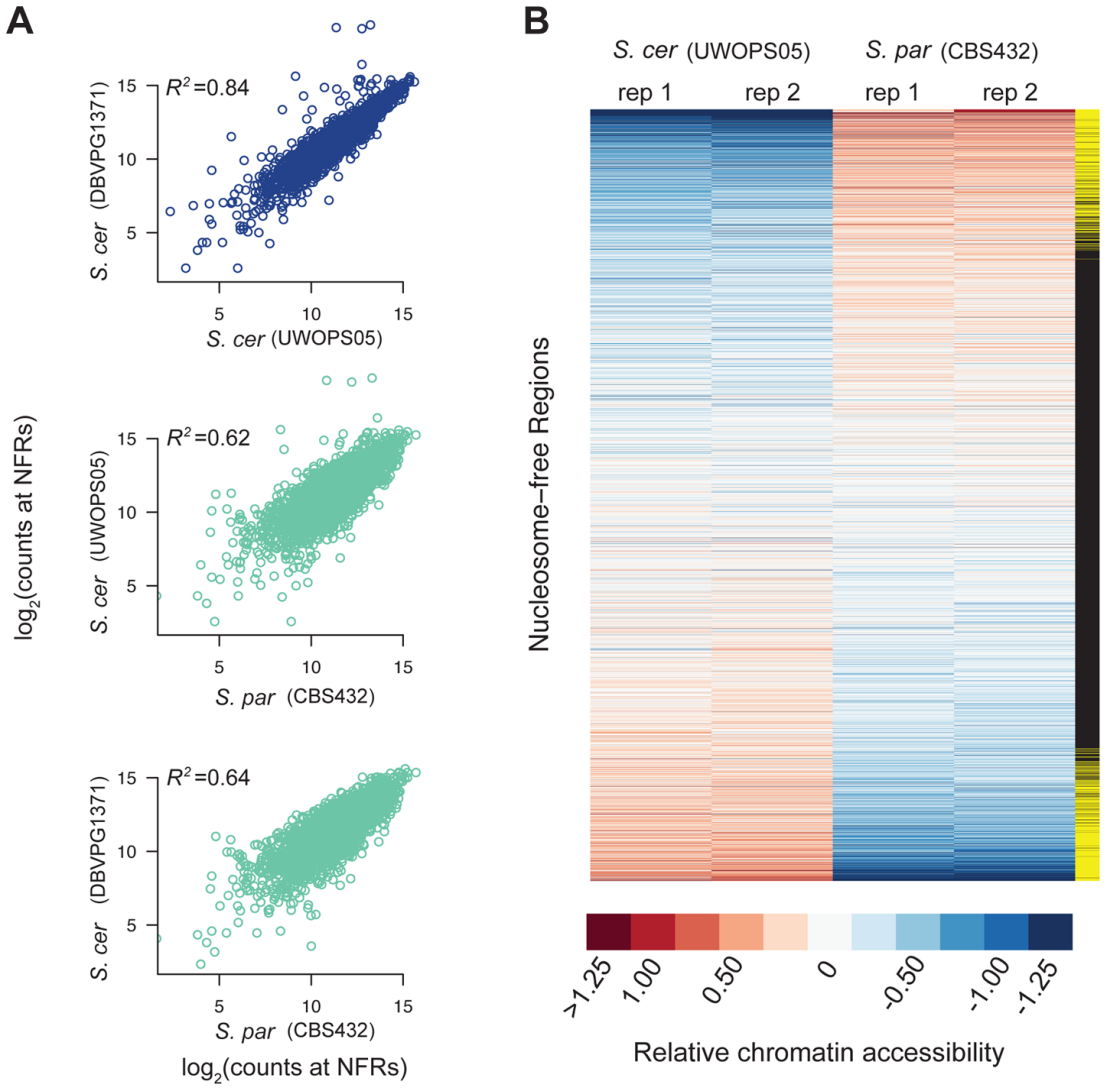
Patterns of chromatin accessibility within and between *S. cerevisiae* and *S. paradoxus*. A. Scatterplots of relative chromatin accessibility between *S. cerevisiae* strains DBVPG1373 and UWOPS05_217_3 (top), *S. cerevisiae* strain UWOPS05_217_3 and *S. paradoxus* strain CBS432 (middle), and *S. cerevisiae* strain DBVPG1373 and *S. paradoxus* strain CBS432 (bottom). Note, comparisons within and between species are shown as blue and light green, respectively. B. Heatmap representation of chromatin accessibility at all NFRs in *S. cerevisiae* strain UWOPS05_217_3 versus *S. paradoxus* strain CBS432. Each row is a NFR, and columns are the two biological replicates of *S. cerevisiae* strain UWOPS05_217_3 and *S. paradoxus* strain CBS432. Rows are sorted by average difference in signal at NFRs between *S. cerevisiae* and *S. paradoxus*. The far right column indicates if the difference in chromatin accessibility between species is significant (yellow rectangles).

Next, we tested each of the 3,498 NFRs for differences in chromatin accessibility between the two parental haploid species, *S. cerevisiae* and *S. paradoxus*, and used the R package DESeq to test for significant differences. We found 947 NFRs showed significant differences in FAIRE signal (FDR = 0.05, Figure 1, see Methods). Furthermore, by analyzing the distribution of *p-*values [42], we estimate that π_0_ (the proportion of NFRs with no differences in chromatin accessibility) is 0.53, suggesting that 47 percent of NFRs are differentially accessible between species. These 947 NFRs were upstream of 1,149 distinct genes and on average resulted in a 2.17-fold difference in FAIRE signal between the two species. 483 of the NFRs showed higher accessibility in UWOPS05_217_3, while 464 NFRs showed higher accessibility in CBS432. We carried out a test for GO enrichment at the genes downstream of differentially accessible peaks and found that several GO biological process terms were enriched compared to the genome as a whole (corrected *p*<0.05), specifically intracellular transport, protein localization, protein transport, and establishment of protein localization [43].

To assess the robustness of these results, we also generated FAIRE-Seq data for a second strain of *S. cerevisiae* (DBVPG1373, a wine strain). Divergence at synonymous sites between these species is estimated to be 0.29 [37]. Levels of chromatin accessibility in NFRs were highly similar between the two *S. cerevisiae* strains (Figure 1; R^2^ = 0.84; *p*<2.2×10^−16^), and of similar magnitude between species (Figure 1; mean R^2^ = 0.63; *p*<2.2×10^−16^). Similarly, of the 947 NFRs that showed differential accessibility between UWOPS05_217_3 and CBS432, 515 were also significantly different between DBVPG1373 and CBS432. Thus, patterns of chromatin accessibility are highly reproducible between genetically diverse strains of *S. cerevisiae* and *S. paradoxus*.

### Genetic architecture of chromatin differences

**Figure 2:**
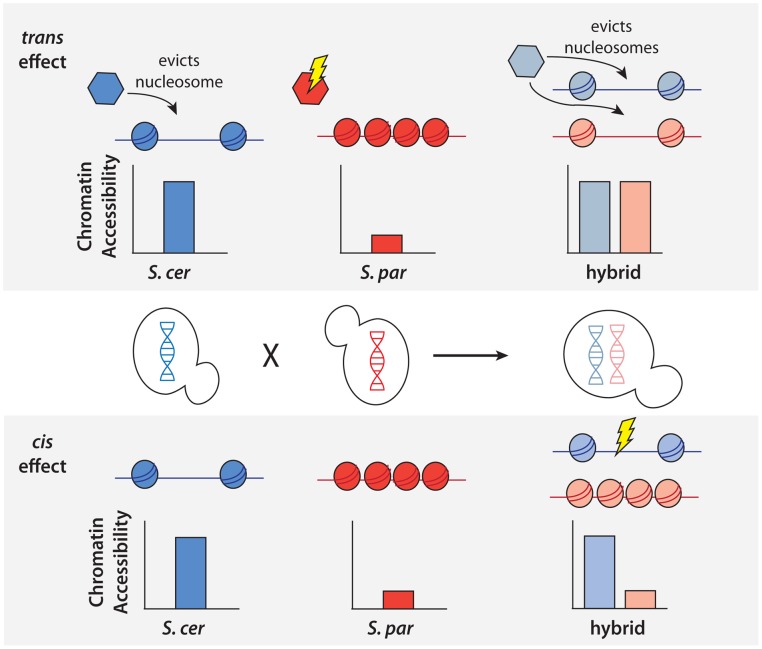
Schematic of approach to detect *cis* and *trans* effects on chromatin accessibility. Top, an example of a NFR showing only a *trans* effect on chromatin accessibility. A *trans* effect is detected as a case where there is a difference in chromatin accessibility between the two parental haploid species, but there is no difference in chromatin accessibility between the two alleles in the hybrid. As shown above, this could be explained by a case where a nucleosome remodeler (shown as a hexagon) acts to evict nucleosomes and increase accessibility in *S. cerevisiae*, but a mutation in *S. paradoxus* has rendered it inactive and it is unable to evict the nucleosomes. In the diploid hybrid, the chromatin remodeler from *S. cerevisiae* is able to evict nucleosomes from both the *S. cerevisiae* and *S. paradoxus* chromosomes. Bottom, an example of a NFR showing only a *cis* effect on chromatin accessibility. A *cis* effect is detected as a difference between the accessibility detected between the two alleles in the diploid, and the lack of a *trans* effect is shown by the same difference being detected between the parental species. In this case, there has been a mutation at the NFR on the *S. cerevisiae* allele, leading to a difference in the number of nucleosomes binding in the region.

**Figure 3:**
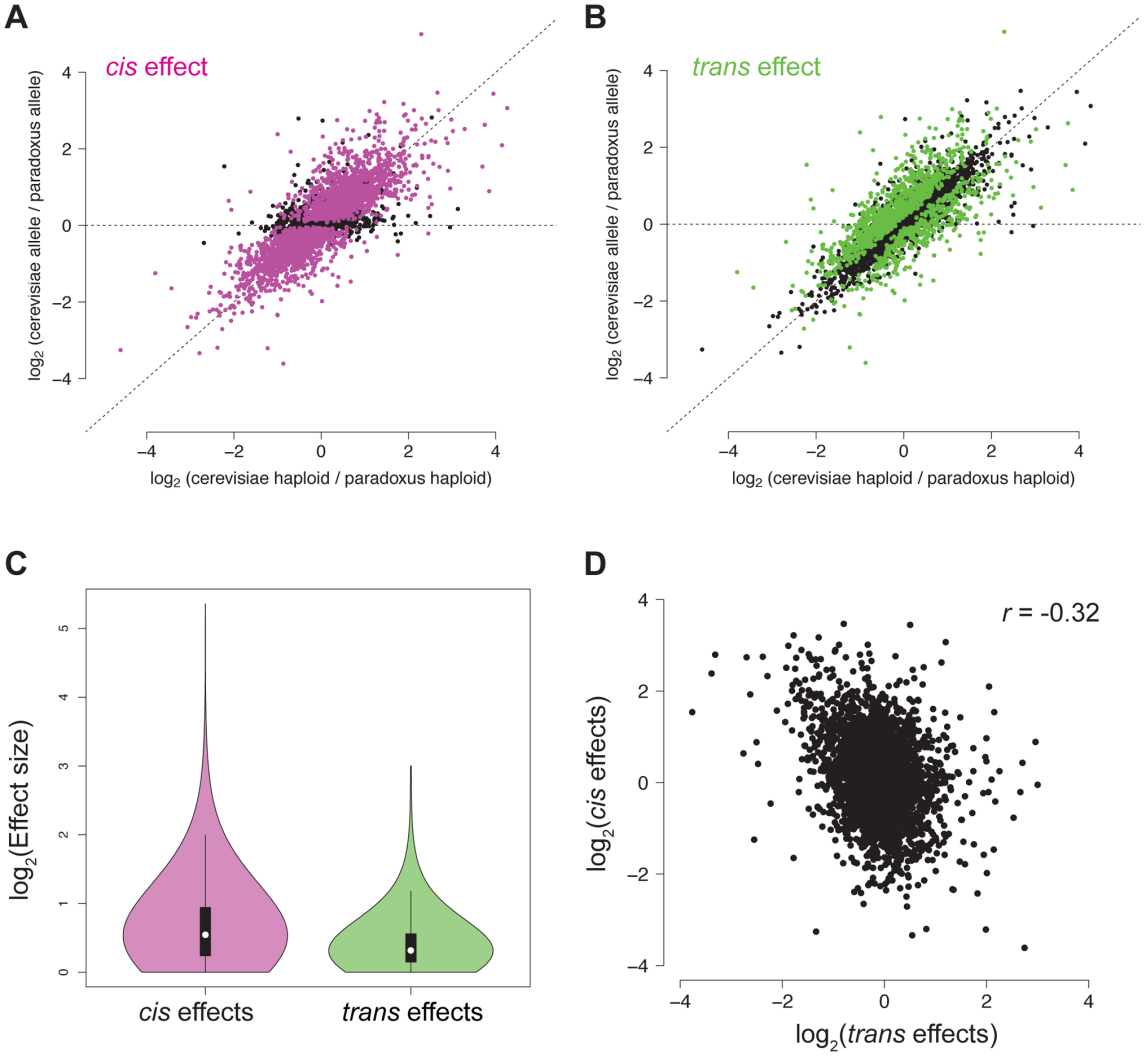
*Cis* and *trans* effects on chromatin accessibility. A. For each NFR, the relative chromatin accessibility in the haploid is plotted versus the relative chromatin accessibility in the diploid. NFRs with a significant *cis* effect are shown in pink. B. Reproduction of the plot from (A), but NFRs with a significant *trans* effect are shown in green. C. Violin plot showing the effect size distribution of *cis* and *trans* effects. D. Scatter plot of relative *cis* and *trans* effect sizes. Positive effects indicate higher accessibility in *S. cerevisiae* and negative effects indicate higher accessibility in *S. paradoxus*.

To better understand the genetic architecture of the widespread differences in chromatin accessibility observed between *S. cerevisiae* and *S. paradoxus*, we developed novel statistical tests for the presence of *cis* and *trans* effects (see Methods; Figure 2). Simulations showed that these tests had high power and maintained correct false positive rates over a range of parameters (see Methods; Table S1). Briefly, we tested for allele-specific chromatin accessibility within the hybrid to identify *cis* effects and tested for differences between the ratio of chromatin accessibility in the two parental species and the ratio of chromatin accessibility observed in the hybrid to identify *trans* effects (Figure 2). Over 99% of all NFRs identified in the parental strains contained one or more variants (median = 32) and could therefore be assessed for *cis* and *trans* effects. We identified 2,256 NFRs showing a significant *cis* effect (posterior probability >0.95, see Figure 3A) and 1,020 NFRs showing a significant *trans* effect (posterior probability >0.95, see Figure 3B). Interestingly, 782 NFRs showed both significant *cis* and significant *trans* effects. *Cis* effects were both more numerous as well as of greater magnitude on average compared to *trans* effects (1.8 and 1.6-fold difference in chromatin accessibility for *cis* and *trans* effects, respectively; Mann Whitney test, *p*<2.2×10^−16^, Figure 3C). Strikingly, we found that *cis* and *trans* effects were negatively correlated (r = −0.32, *p*<1×10^−16^), which suggests a widespread role for compensatory evolution to stabilize chromatin structure (Figure 3D).

### Disrupted motifs are associated with *cis* effects

To test the hypothesis that *cis*-acting chromatin QTL result from variation in regulatory motifs, we identified motifs independently in the two species and computationally inferred whether sequence differences abrogated motif usage. Specifically, we define disrupted motifs as those that were called in only one of the two species (see Methods). Disrupted motifs were strongly enriched in NFRs with significant *cis*-acting chromatin QTL (*p* = 2.4×10^−7^). We also found that overall nucleotide divergence was higher at NFRs with significant *cis* effects compared to regions without significant *cis* effects (Mann Whitney test, *p* = 3.48×10^−6^). Note, this observation parallels previous findings that polymorphism is higher for genes that show significant allele-specific expression in *S. cerevisiae* hybrids [44].

**Figure 4:**
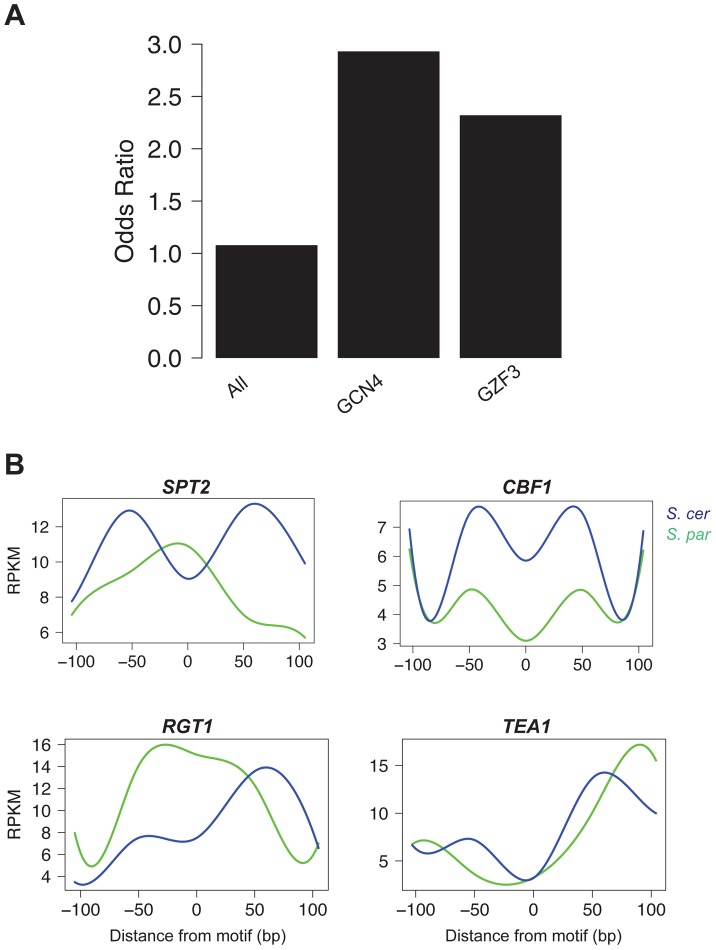
Motifs contributing to *cis* and *trans* effects. A. The odds ratio of observing a disrupted motif compared to a non-disrupted motif in NFRs with a significant *cis* effect. Odds ratios are shown for all motifs, as well as the two individual motifs (*GCN4* and *GZF3*) that were found to be significant by permutations (FDR = 0.10). B. Pattern of accessibility for four motifs found within *trans* effect NFRs that vary between *S. cerevisiae* and *S. paradoxus*.

We next asked if any of the 106 motifs were overrepresented for being disrupted in the set of significant *cis*-acting chromatin QTL. We found two overrepresented motifs, *GCN4* and *GZF3* (FDR = 0.10; Figure 4A). *GCN4* is an activator of amino acid biosynthetic genes, which itself is a tightly regulated pathway [45]. *GZF3* is a negative regulator of nitrogen catabolic gene expression [46]. While it is not immediately clear why disruption of these two genes is associated with changes in chromatin structure, it is interesting that both play an important role in metabolism, which is a highly regulated process.

### Differential footprints for certain DNA binding factors found at *trans* effects loci

To identify factors contributing to *trans* effects, we searched for cases where there was no disruption to the motif but the occupancy of the site changed between species. Such patterns could result from mutations that either alter the binding specificity of a *trans*-acting regulatory protein or change its regulation. We used the FAIRE data surrounding each motif to determine occupancy, analogous to a DNase I footprint [39]. We then tested whether there was a significant difference in the pattern of occupancy between species by fitting splines to the mean occupancy across conserved sites in *trans* regions and testing whether the splines were significantly different in a 100 bp window surrounding the motif using bootstrapping (see Methods). We identified four motifs whose pattern of occupancy had significantly (*p*<0.05) changed between species (Figure 4B). *SPT2*, a transcription factor that interacts with histones and the SWI/SNF complex, showed a clear footprint in *S. paradoxus*, but nearly the opposite pattern in *S. cerevisiae*, implying decreased occupancy in *S. cerevisiae* at these *trans* regions. Similarly, *TEA1*, a Ty enhancer activator, and *RGT1*, a glucose-responsive transcription factor, showed increased occupancy in *S. paradoxus*. Conversely, *CBF1*, a centromere binding factor also involved in stress response, showed higher FAIRE signals in *S. paradoxus* than *S. cerevisiae*, implying increased occupancy in *S. cerevisiae*.

### Effects on gene expression

**Figure 5:**
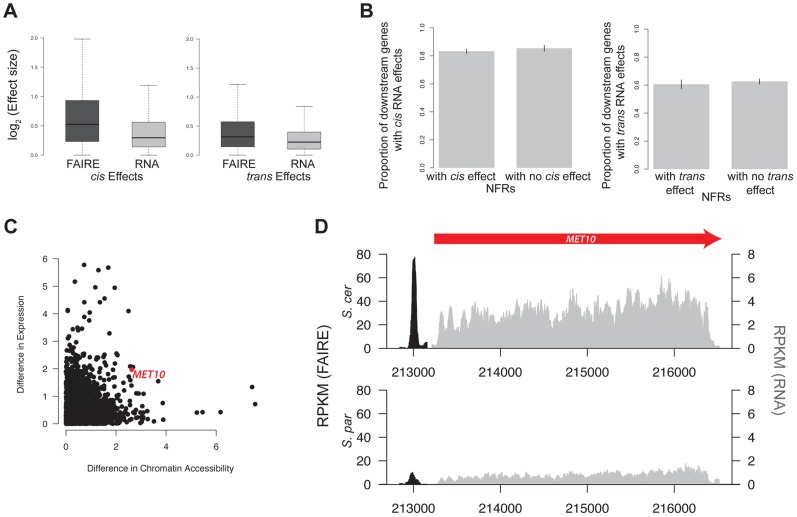
Gene expression and chromatin accessibility. A. Boxplot of log_2_(effect size) of both *cis* and *trans* effects for FAIRE (dark grey) and RNA (light grey). B. Barplot of the percentage of genes with significant *cis* effects in RNA that are downstream of NFRs with and without *cis* effects (left). Barplot of the percentage of genes with significant *trans* effects in RNA that are downstream of NFRs with and without *trans* effects (right). C. Scatterplot of the log_2_(absolute value of the difference in chromatin accessibility between the two species) vs log_2_(absolute value of the difference in expression between the two species. The red dot indicates data from the *MET10* gene, whose FAIRE-Seq and RNA data are shown in panel D. For clarity, the FAIRE-Seq data is only shown in a 100 bp window on either side of the NFR. FAIRE signal is shown in black, and RNA signal is shown in grey.

To examine the relationship between differences in chromatin accessibility and transcriptional divergence between *S. cerevisiae* and *S. paradoxus*, we performed RNA-Seq on the haploid parents and interspecific hybrid and tested for the *cis* and *trans* effects on gene expression values. Out of the 4,899 genes that could be aligned between species, 4,181 exhibited significant *cis* effects and 3,117 showed significant *trans* effects. Overall, *cis* and *trans* effects on gene expression levels were smaller than those on chromatin accessibility, (Spearman rank-sum test, *p*<2.2×10^−16^ for both *cis* and *trans* effects, Figure 5A).

We next tested whether genes with a significant *cis* or *trans* effect in chromatin were more likely to have a significant *cis* or *trans* effect in transcript abundance. Specifically, we divided genes into categories of those downstream of an NFR with a *cis* effect on chromatin versus those downstream of an NFR without a *cis* effect on chromatin. We then compared the percentage of genes showing *cis* effects on RNA in these two categories. Surprisingly, we did not find evidence that *cis* or *trans* effects in NFRs were more likely to be upstream of *cis* or *trans* effects on RNA, as would be expected if there was a simple correspondence between *cis* and *trans* effects in NFRs and RNA (see Figure 5B, Table S2). This was true even when using varying cutoffs for the *cis* and *trans* effects, including ones that took into account the magnitude of effect sizes (Table S2).

The relationship of *cis* and *trans* effects observed in gene expression and chromatin structure may be complicated by differences in statistical power. For example, 85% of all genes show significant *cis* effects on RNA. Thus, even if *cis* effects in NFRs are not more likely to be found upstream of *cis* effects on RNA, they could still contribute to gene expression variation between *S. cerevisiae* and *S. paradoxus*. To this end, we assessed whether expression differences between species could be modeled as a function of the *cis* and *trans* effects found upstream of each gene. Specifically, we fit the simple linear model: expression difference = Intercept + *cis* effect + *trans* effect + *cis* * *trans* effect + error, using the lm function in R. We found that both *cis* effects and *trans* effects on chromatin were significantly related to expression differences between species (*p* = 0.002, *p* = 4.18×10^−5^ respectively) though they explained a very small proportion of the total variance in expression between species (0.8% combined). The interaction term of *cis* and *trans* effects was not significant (*p*>0.05). The motif for GZF3, which is significantly overrepresented in *cis* NFRs, was overrepresented in *cis* NFRs upstream of genes with *cis* effects on RNA.

Finally, we found no significant correlation between the magnitude of differences in chromatin accessibility and differences in gene expression between the parental species (Spearman rank-sum test, *p* = 0.11, Figure 5C). However, for a subset of NFRs, differences in chromatin accessibility and gene expression do appear to be highly correlated. To identify these regions, we compared the log_2_(*S. paradoxus*/*S. cerevisiae*) for NFRs and gene expression at downstream genes and identified those whose absolute value of the difference between the two ratios was less than 0.25. We identified 701 such regions; one example is shown in Figure 5D.

## Discussion

The ability to assay chromatin accessibility at high-resolution and on a genome-wide scale has enabled comprehensive insights into the structure and function of chromatin in many cell types, developmental stages, and organisms. Here, we were particularly interested in the evolutionary dynamics of changes in chromatin accessibility between two closely related yeast species. Broad-scale patterns of chromatin accessibility have been well conserved between *S. cerevisiae* and *S. paradoxus* (Figure 1), but superimposed on this background of conservation, we estimate that nearly 50% of NFRs exhibit differential accessibility.

To better understand the relative contributions of *cis* and *trans* effects on differences in chromatin accessibility observed between *S. cerevisiae* and *S. paradoxus*, we developed novel statistical methods to analyze FAIRE-Seq data from diploid hybrids. Similar to previous findings on RNA levels [17], [21], [22], [25], differences in chromatin accessibility are caused by changes both in *cis* and in *trans.* In our data, *cis* effects were of greater magnitude and were more abundant. Recently, Lee et al. performed a study similar to ours and assessed *cis* and *trans* effects on chromatin structure in a cross between two strains of *S. cerevisiae* [34]. In contrast to our observations, they found that *trans* QTL were more pervasive than *cis* QTL (92.1% of associations versus 7.9% of associations) [34]. We hypothesize that these disparate observations are primarily the consequence of differences in the evolutionary trajectory of chromatin accessibility QTL in within versus between species data. In particular, *trans*-acting chromatin QTL are likely to be subject to more intense purifying selection due to their potential pleiotropic effects, and tend to be eliminated over longer time periods [47]. This hypothesis is consistent with findings for expression QTL studies, which showed that *trans*-eQTL were more common within species and *cis*-eQTL were more common between species [22], [23]. Consistent with this hypothesis, we found that *cis* and *trans* effects were significantly negatively correlated, indicating that chromatin accessibility in each species is subject to stabilizing selection and perturbations of chromatin structure are, on average, deleterious.

We estimated *cis* and *trans* effects for both chromatin accessibility and gene expression levels. Unexpectedly, the presence of *cis* or *trans* effects on chromatin accessibility in NFRs was not significantly associated with *cis* or *trans* effects on RNA. In other words, gene expression levels with significant *cis* or *trans* effects were not more likely to have an NFR with significant *cis* or *trans* effects on chromatin accessibility. Thus, it appears that many of the changes in chromatin accessibility in NFRs between *S. cerevisiae* and *S. paradoxus* do not necessarily have transcriptional consequences. One factor that may contribute to this observation is that compensatory changes downstream of chromatin accessibility, such as mutations that influence mRNA stability, may evolve to maintain levels of gene expression. In addition, many changes in chromatin accessibility may simply be functionally benign.

Furthermore, an important caveat is that our data was obtained from a single environmental condition, and it is plausible that stronger correlations between chromatin and gene expression QTL may exist when analyzing data from either a different environment or across multiple environments. Nonetheless, the lack of a clear relationship between chromatin and gene expression QTL in our data is interesting in light of recent observations from the ENCODE Project [48] that have found a large proportion of the human genome has reproducible biochemical activity. Our results suggest caution in assuming all, or perhaps even most, of such sequences are functionally important.

## Materials and Methods

### Strain growth, FAIRE, and RNA-Seq

65 ml of each of 2 biological replicates of the *S. paradoxus* strain CBS432 and the two *S. cerevisiae* strains DBVPG1373 and UWOPS05_217_3 were grown to mid-log phase. 15 ml were used for RNA-seq and 50 ml were used for FAIRE. We performed FAIRE as described in Simon et al. [40], with some modification. The cells were fixed with 1% formaldehyde for 35 minutes with mixing. Cells were sonicated using the Fisher Scientific Sonic Dismembrator Model 100 for three cycles of 15 one-second bursts with 1 second rest in between, keeping the cells on ice for at least 30 seconds between cycles. The remainder of the protocol was followed as in Simon et al. [40]. RNA isolation was performed using the hot phenol protocol [49], and RNA was treated with Turbo DNAse before library construction.

### Library construction and sequencing

Libraries were constructed for the FAIRE samples using the Illumina TruSeq DNA kit, starting with approximately 200 ng FAIRE DNA, following their standard kit protocol but omitting the fragmentation step. RNA libraries were prepared using the Illumina TruSeq RNA kit, following their standard protocol. Libraries were pooled into two lanes, one for the FAIRE samples and one for the RNA samples, and were sequenced on the HiSeq 2000. Raw sequence data and processed files are available at the GEO database with accession number GSE55717.

### Read mapping

Reads were mapped to genomes assembled in Skelly et al. [50] for the *S. cerevisiae* haploid samples using bwa and samtools [51], [52]. For the *S. paradoxus* strain CBS432, we used the last updated reference version from the SGRP [53]. For the diploid samples, we mapped to a combined FASTA containing both genomes. We tested whether mapping to each genome separately for the diploid samples resulted in increased mapping; it did not. For the diploid samples, we generated simulated reads and mapped to the combined FASTA. For all further analyses, we restricted analysis to NFRs for which greater than 90 percent of simulated reads mapped back to the correct region. We also sequenced a genomic DNA sample. We also filtered out NFRs where the absolute value of the log_2_(ratio of reads between the two species) for the genomic DNA was greater than 0.3.

### Identifying NFRs

We identified NFRs as follows: specifically, starting at the beginning of the coding region of the gene, we looked for the peak of chromatin accessibility within 300 bp upstream of the start codon. We then defined the edges of the NFR as the base-pair after which at least 3 bases had had a chromatin accessibility count of less than 10. We did this separately for each biological replicate and each species. For each gene separately, we then merged NFRs if they were within 200 bp.

### Filtering NFRs and genes

In order to convert between the two species coordinates, we created a multiple alignment between the two species using LASTZ and TBA [54], [55]. We inferred scoring parameters using the two species of interest. Using this multiple alignment, we then converted the NFRs called in CBS432 to *S. cerevisiae* coordinates, and found the union of all NFRs called across the samples. We used this union of NFRs for further tests. We also filtered the NFRs based on a reciprocal alignment filter, where we required that NFRs align to only one region in the other species, based on the multiple alignment. This allowed us to filter out regions with duplications or deletions between the two species.

### Identifying differentially accessible NFRs

Using samtools, we summed the count of reads mapping in each species across each NFR or gene in both biological replicates. Note that we did this in the native coordinates for each species, filtering out sites that were called as indels in the multiple alignment. We then used the R package DESeq [56] to assess differential FAIRE signal between species. This method takes into account biological replicates, and models the count distribution using a negative binomial distribution. We used the R package qvalue [42] to estimate q-values. We used a significance threshold of FDR = 0.05 unless otherwise noted.

### Statistical model to detect *trans* effects

If differences in chromatin accessibility between *S. cerevisiae* and *S. paradoxus* are due to *trans*-acting factors, the relative chromatin accessibility in the haploid parents will be different than the relative chromatin accessibility in the diploid hybrid (Fig. 2). We leveraged the FAIRE-Seq data to detect differences in the relative levels of chromatin accessibility between F_1_ hybrids and the parental species. Specifically, let *N_c_* and *N_p_* be the total number of reads across the genome mapping to polymorphic sites in the *S. cerevisiae* and *S. paradoxus* haploid parents, respectively. For a particular locus *j*, *Y_c_* and *Y_p_* denote the observed number of reads mapping to *S. cerevisiae* and *S. paradoxus*, respectively. Then assume: *Y_c_*|*r_c_*∼Binomial (*N_c_*, *r_c_*) and *Y_p_*|*r_p_*∼Binomial(*N_p_*, *r_p_*), where *r_c_* and *r_p_* denote the probabilities of observing a read mapping to *S. cerevisiae* or *S. paradoxus* for a particular locus, respectively. Since *N_c_* and *N_p_* are large, and *r_c_* and *r_p_* are small, we can approximate these binomials by Poissons to give: *Y_c_*|*r_c_*∼Poisson(*N_c_ r_c_*) and *Y_p_*|*r_p_*∼Poisson(*N_p_ r_p_*).

We define *θ_P_ = r_c_*/*r_p_* to be the ratio of these probabilities in the parents and *R* = *N_c_*/*N_p_* to be the ratio of the total numbers of reads in each parent. Then, *Y_c_*|*Y_c_*+*Y_p_*, *s_c_*∼Binomial(*Y_c_*+*Y_p_*, *s_c_*), where *s_c_* = *N_c_r_c_*/(*N_c_r_c_*+*N_p_r_p_*) = *Rθ_P_*/(*Rθ_P_*+1) is the probability of observing a read map to *S. cerevisiae*, without adjusting for differences in the total number of reads mapping to each species. We can thus write log(*S_c_*/1−*S_c_*) = log *R*+log *θ_P_*, such that *θ_P_* is the odds of observing a read map to *S. cerevisiae* compared to *S. paradoxus* for a particular locus in the haploid parents, adjusted for differences in the total number of reads mapping to each species.

For the diploid hybrid, let *Z_c_* and *Z_p_* denote the number of reads mapping to *S. cerevisiae* and *S. paradoxus* SNPs within locus *j*, respectively. Thus, *Z_c_*|*Z_c_*+*Z_p_*, *p_c_*∼Binomial(*Z_c_*+*Z_p_*, *p_c_*), where *p_c_* is the probability of observing a read map to the *S. cerevisiae* allele for a particular locus. The odds of observing a read map to *S. cerevisiae* in the hybrid for a particular gene is *θ_H = _p_c_*/(1−*p_c_*). In the following, let *Y_cj_*, *Y_pj_*, *Z_cj_*, and *Z_pj_* represent the data as defined above, but with *j* = [1], [2] indexing biological replicate.

Thus, the locus specific models are:

*Y_cj_*|*Y_cj_*+*Y_pj_*, *s_cj_*∼Binomial(*Y_cj_*+*Y_pj_*, *s_cj_*),

*Z_cj_*|*Z_cj_*+*Z_pj_*, *p_cj_*∼Binomial(*Z_cj_*+*Z_pj_*, *p_cj_*)

logit *s_cj_* = log *R_j_*+log *θ_P_*+*δ_j_*

logit *p_cj_* = log *θ_P_*+Δ+*ε_j_*

where *R_j_* = *N_cj_*/*N_pj_*, *δ_j_*∼N(0, σ^2^) and *ε_j_*∼N(0, σ^2^) represent random effects that allow for excess-binomial variation. Here, Δ is the parameter of interest and provides an estimate of the difference between log(*θ_P_*) and log(*θ_H_*), as described above. The above framework is an example of a generalized linear mixed model (GLMM) and we used a Bayesian approach to inference with relatively flat hyperpriors. One computationally intensive method for summarizing the posterior would be Markov chain Monte Carlo (MCMC) but the integrated nested Laplace approximation (INLA) as described in [57] provides an efficient alternative for GLMMs [58]. We used the R implementation of INLA to estimate Δ. We examined a 95% posterior interval estimate for Δ and recorded whether this interval contained 0 or not. If the interval does not contain 0 it indicates that chromatin accessibility differs.

### Statistical model to detect *cis* effects

To detect *cis* effects, we developed a model to test for differential accessibility between alleles within the diploid hybrid. Let *Z_cj_*, and *Z_pj_* represent the data as defined above. We can therefore write:

*Z_cj_*|*Z_cj_*+*Z_pj_*, *p_cj_*∼Binomial(*Z_cj_*+*Z_pj_*, *p_cj_*)

logit *p_cj_* = log *θ_H_*+*ε_j_*

with *ε_j_*∼N(0,σ^2^) representing random effects that allow for excess-binomial variation. In this model, *θ_H_* is the parameter of interest and provides an estimate of the odds of a read mapping to the *S. cerevisiae* allele compared to the *S. paradoxus* allele in the diploid hybrid for a particular gene. We again used the R program INLA to estimate the posterior for log(*θ_H_*) and in particular examine whether the 95% posterior interval estimate contains 0.

### Simulations

We carried out extensive simulations to evaluate the operating characteristics of our model. Specifically, for the *trans* model, we set the total number of reads mapping to polymorphic sites for species 1 (*N_c1_*) equal to 5×10^6^, and drew the total number of reads mapping to polymorphic sites for the other species and replicate from a normal distribution with mean *N_c1_* and standard deviation *N_c1_*. We then drew the value for *r_c_*, the probability of a read mapping to *S. cerevisiae* for a particular locus from an exponential distribution with rate 10,000. For *N_c1_* = 5×10^6^, this results in a mean of 500 reads mapping to a locus, with most having less than 500 reads, consistent with the observed data. We drew the value for *r_p_*, the probability of a read mapping to *S. paradoxus* for a particular locus, from a normal distribution with mean *r_c_* and standard deviation *r_c_* and took the absolute value to ensure *r_p_* was greater than zero. Using these values, we derived *Y_c_* and *Y_p_*, the number of reads mapping to *S. cerevisiae* and *S. paradoxus*, respectively, for a particular locus, for two biological replicates as specified by the model. For *Z_c_* and *Z_p_*, the number of reads mapping to the *S. cerevisiae* and *S. paradoxus* alleles in the hybrid summed across polymorphic sites in a particular locus, we either derived these using the same *r_c_* and *r_p_* values as above, to simulate a locus which showed no *trans* effect, or we set the value of log_2_(*θ_P_)−*log_2_(*θ_H_*) equal to 0.1, 0.5, or 0.8, to simulate a locus with a *trans* effect. Note, this spans the range of detected *trans* effects. For 100 replicates, we simulated a collection of 6000 loci, 5000 of which did not show a *trans* effect and 1000 or which did show a *trans* effect. For each of the 100 replicates, we then used the method described above to test whether the 95% posterior interval estimate for Δ for each locus contained zero.

To evaluate the *cis* test, we again started with the same values for the total number of reads. To simulate a locus with no *cis* effect, we set the value of log_2_(*Z_c_*/*Z_p_*) equal to zero, and to simulate a locus with a *cis* effect, we set the value of log_2_(*Z_c_*/*Z_p_*) equal to 0.1, 0.5, or 0.8. Again, for 100 simulations, we simulated a collection of 6000 loci, 5000 showing no *cis* effect and 1000 showing a *cis* effect. For each simulated set of loci, we then used the statistical method above to test whether the 95% posterior interval estimate for log(*θ_H_*) for each locus contained zero to test for a significant *cis* effect.

We found that the false discovery rate for both *cis* and *trans* based on a test based on a 95% interval was 0.05. Moreover, we found that the *trans* test has reduced power compared to the *cis* test, as expected because there were more parameters that could vary across biological replicates. However, with an effect size = 0.5 for both the *cis* and *trans* tests, there was significant power to detect the *cis* or *trans* effects (Table S1).

### Motif analysis

We called motifs separately in both species, using MEME, using their standard p value cutoff of *p*<10^−4^ [59]. This results in the same cutoffs used for both species. Motifs that were not called in both species were considered polymorphic. We filtered out motifs where the polymorphism was due to indels in order to mitigate alignment errors. The motif calls used for this analysis are available as supplementary data on our website (http://akeylab.gs.washington.edu/downloads.shtml). We compared the proportion of disrupted motifs (those that were called in only one species) in *cis* NFRs to non-*cis* NFRs using the Fisher exact test. We determined significance by permutations; we permuted the assignment of *cis* or not *cis* NFRs 1000 times and obtained p values from the permutations. We then used the positive False Discovery Rate approach to determine significance [42].

### Occupancy at *trans* NFRs

We obtained the RPKM in a 200 bp window surrounding motifs that were conserved across species in *trans* NFR regions for each of the two species. We filtered out motifs that did not have at least five instances of conserved motifs. We fit a cubic smoothing spline to the mean coverage using the R function spline. We then bootstrapped the data 1000 times by resampling from the motifs for each species. At five bp intervals across the region, we then tested whether the coverage was significantly different between the species, using the confidence intervals obtained from the bootstrapping. We then manually inspected the significant motifs (*p*<0.05) to identify those which appeared to affect the FAIRE signal at or the near the motif.

## Supporting Information

Figure S1 Enrichment of FAIRE signal in NFRs and intergenic regions. RPKM for *the S. cerevisiae* strain UWOP05_217_3 and the *S. paradoxus* strains CBS432 is shown in three types of regions, nucleosome-free regions (NFRs), intergenic regions, and genic regions.
(PDF)

Table S1 Power and false discovery rate for *cis* and *trans* tests from simulations.
(DOCX)

Table S2 Summary of different criteria used to investigate the relationship between chromatin and gene expression QTL
(DOCX)
