# Supplementary figures and images for: Evolution and Genetic Architecture of Chromatin Accessibility and Function in Yeast
Source: PLoS Genet. 2014 Jul 3;10(7):e1004427. doi: 10.1371/journal.pgen.1004427 (PMC4081003; doi:10.1371/journal.pgen.1004427)

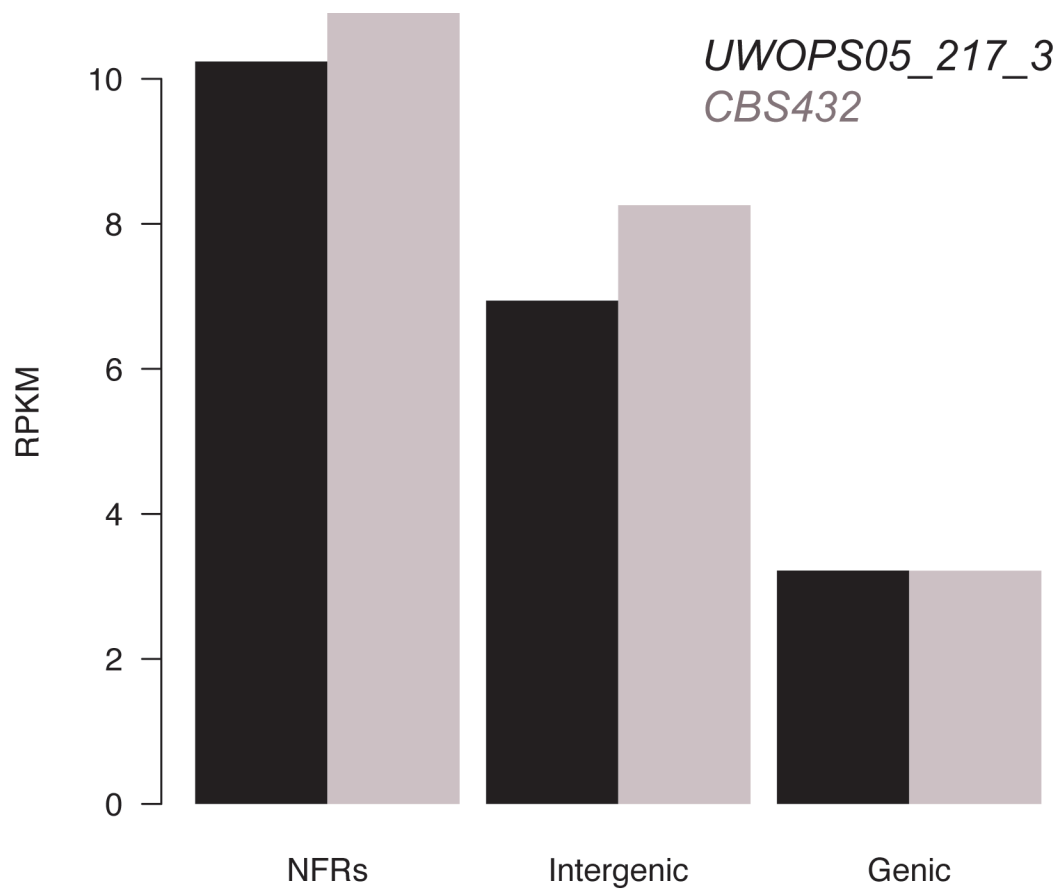

Supplement: Figure S1 — Enrichment of FAIRE signal in NFRs and intergenic regions. RPKM for the S. cerevisiae strain UWOP05_217_3 and the S. paradoxus strains CBS432 is shown in three types of regions, nucleosome-free regions (NFRs), intergenic regions, and genic regions. (PDF) [file pgen.1004427.s001.pdf]
