# Supplementary material for: Evolution and Genetic Architecture of Chromatin Accessibility and Function in Yeast
Source: PLoS Genet. 2014 Jul 3;10(7):e1004427. doi: 10.1371/journal.pgen.1004427 (PMC4081003; doi:10.1371/journal.pgen.1004427)
Supplement: Table S1 — Power and false discovery rate for cis and trans tests from simulations. (DOCX) [file pgen.1004427.s002.docx]

**Table S1. Power and false positive rate for *cis* and *trans* tests.**

| **Type of test** | **Effect size** | **Power (at posterior probability=0.95)** | **False positive rate (at posterior probability=0.95)** |
| --- | --- | --- | --- |
| *cis* | 0.1 | 0.32 | 0.05 |
| *cis* | 0.5 | 0.92 | 0.05 |
| *cis* | 0.8 | 0.97 | 0.05 |
| *trans* | 0.1 | 0.18 | 0.05 |
| *trans* | 0.5 | 0.81 | 0.05 |
| *trans* | 0.8 | 0.91 | 0.05 |
