# Supplementary material for: Evolution and Genetic Architecture of Chromatin Accessibility and Function in Yeast
Source: PLoS Genet. 2014 Jul 3;10(7):e1004427. doi: 10.1371/journal.pgen.1004427 (PMC4081003; doi:10.1371/journal.pgen.1004427)
Supplement: Table S2 — Summary of different criteria used to investigate the relationship between chromatin and gene expression QTL (DOCX) [file pgen.1004427.s003.docx]

| **Criteria for calling *cis* effects** | ***cis* effect on NFR** | | **No *cis* effect on NFR** | | **OR** | ***p-value*** |
| --- | --- | --- | --- | --- | --- | --- |
|  | *cis* effect on RNA | No *cis* effect on RNA | *cis* effect on RNA | No *cis* effect on RNA |  |  |
| PP > 0.95 | 1790 | 358 | 971 | 165 | 0.85 | 0.12 |
|  |  |  |  |  |  |  |
| PP > 0.95 &  log_2_(magnitude of NFR effect) > 1 | 526 | 122 | 2235 | 401 | 0.77 | 0.03 |
|  |  |  |  |  |  |  |
| PP > 0.95 & log_2_(magnitude of NFR and RNA effect) >1 | 185 | 1312 | 208 | 1579 | 1.07 | 0.55 |
|  |  |  |  |  |  |  |
| PP > 0.998 | 1125 | 459 | 1244 | 459 | 0.90 | 0.17 |
|  |  |  |  |  |  |  |
| PP > 0.998 & log_2_(magnitude of NFR effect) > 1 | 387 | 166 | 1982 | 749 | 0.88 | 0.23 |
|  |  |  |  |  |  |  |
| PP > 0.998 & log_2_(magnitude of NFR and RNA effect) >1 | 136 | 1073 | 230 | 1875 | 1.02 | 0.91 |
|  |  |  |  |  |  |  |
| PP > 0.95 & log_2_(*trans* effect) <0.10 for RNA | 466 | 1682 | 219 | 917 | 1.16 | 0.11 |

**Table S2. Summary of different criteria used to investigate the relationship between chromatin and gene expression QTL.**

Notes: PP and OR denote posterior probability and odds ratio, respectively.
